# Rapid Evaluation of Spermidine from 12 Bean Cultivars by Direct Real-Time Mass Spectrometry Analysis

**DOI:** 10.3390/molecules23092138

**Published:** 2018-08-25

**Authors:** Tao Wu, Xiaoyu Wu, Xv Yuan, Yi Wang, Wenhua Zhou, Weili Li

**Affiliations:** 1 School of Food and Biotechnology, Xihua University, No. 9999 Hongguang Avenue, Chengdu 610039, China; wutao@mail.xhu.edu.cn (T.W.); wxy501267118@126.com (X.W.); suju980802948@126.com (X.Y.); 2 Xi’an Manareco New Materials Co. Ltd., Xi’an 710077, China; wang.yi@xarlm.com; 3 Key Laboratory of Processed Food for Special Medical Purpose, Central South University of Forestry and Technology, No. 498 Shaoshan Road, Changsha 410004, China; zhowenhua@126.com; 4 College of Food Engineering and Biotechnology, Tianjin University of Science and Technology, Tianjin 300000, China

**Keywords:** spermidine, bean, DART-MS, UHPLC-ESI-QTOF

## Abstract

The routine spermidine (SPD) detection method is time-consuming and laborious due to the lengthy chromatographic separation and/or tedious sample derivatization pretreatment. In this study, direct analysis in real-time ionization mode coupled with mass spectrometry (DART-MS) was developed to rapidly determine the SPD content of 12 bean cultivars. The results were compared in detail with those of the classical UHPLC-ESI-QTOF method. After conducting a series of optimizations, a simple sample extraction procedure employing 80% aqueous methanol, was followed by determination of sample extracts directly without any chromatographic separation or prior derivatization. The validated method showed excellent performance with low limits of detection (LOD of 0.025 mg·kg^−1^) and good recovery rates (102.79–148.44%). The investigation highlighted that the DART-MS method (~1.3 min per three samples) could be used as a high-throughput alternative to the classic UHPLC-ESI-QTOF method (~15 min per three samples).

## 1. Introduction

Spermidine (SPD) is classified as a polyamine due to the number of amino groups in its chemical structure. It displays pleiotropic effects that include antioxidant properties and anti-inflammatory functions as well as improvement of mitochondrial metabolic function [1]. In recent years, the anti-aging activity of SPD has gained increasing attention [2]. Many anti-aging effects of spermidine are associated with the degradation of damaged organelles and recycling of cytoplasmic material via activation of the autophagic machinery [3,4]. Consequently, an extra supply of foods rich in spermidine might have significant health benefits. 

Beans are widely consumed worldwide, especially in Asian countries. Bean seeds contain polyamines such as SPD, spermine, putrescine and cadaverine. In particular, the SPD level of the soybean is considerably higher than that of common cereals, vegetables, or animal food products [5]. This might be due to the high level of arginine, a major precursor of polyamine biosynthesis. Due to the beneficial properties of beans and their worldwide consumption, it is desirable to have a fast, reliable and accurate method of quantifying SPD in food samples.

Various methods to measure the capsaicinoid content have been used by researchers. The determination of polyamines is performed by high-performance liquid chromatography (HPLC), gas chromatography, and capillary electrophoresis. The most common method is HPLC coupled to an ultraviolet detector (UVD) or fluorescence detector (FD). Due to the structure of SPD without any chromophore or fluorophore, the detection of SPD by UVD or FD requires complicated pretreatment steps with derivatization reagents such as 6-aminoquinolyl-*N*-hydroxysuccinimidyl carbamate (AQC) [6], benzoyl chloride [7] or *o*-phthaldialdehyde [8]. Though HPLC is effective when used in a laboratory, the SPD determination procedure is time-consuming due to the lengthy chromatographic separation and sample derivatization process. Moreover, these procedures increase the use of toxic reagents and solvents, which is not environmentally friendly. 

Ambient mass spectrometry (AMS) refers to those techniques that have the capability of both sampling through desorption and ionization under open-air conditions for the subsequent MS analysis. Since first reported, over thirty different techniques have become available [9]. AMS can be classified into three groups based upon the different ionization mechanisms: (1) an ambient gas-, heat- or laser assisted desorption/ionization technique such as atmospheric analysis probe (ASAP) [10] and surface assisted laser desorption ionization mass spectrometry (SALDI-MS) [11] where a solid or liquid sample is ionized at atmospheric pressure; (2) spray or jet ionization techniques such as desorption electrospray ionization (DESI) [12] where charged droplets are produced from an electrospray needle under a high voltage; (3) electric discharge ambient ionization techniques, such as direct analysis by real-time mass ionization (DART) [13] where ions, electrons and metastable atoms are produced using helium/nitrogen and a corona discharge. The advantages of DART-MS versus DESI-MS and other modern rapid mass spectrometry techniques is that they are considered a soft ionization methid and strong fragmentations can rarely be observed [14], which helps the qualitative and quantitative analysis of target components. Therefore, the present study aims to develop a rapid DART-MS method to estimate SPD levels in 12 common bean cultivars without any prior derivatization or chromatographic separation. Furthermore, the measurement conditions were optimized and comprehensively compared with those of the classic UHPLC-ESI-MS method.

## 2. Results and Discussion

### 2.1. Comparison of MS and MS/MS Spectra of SPD Fragments

**Figure 1:**
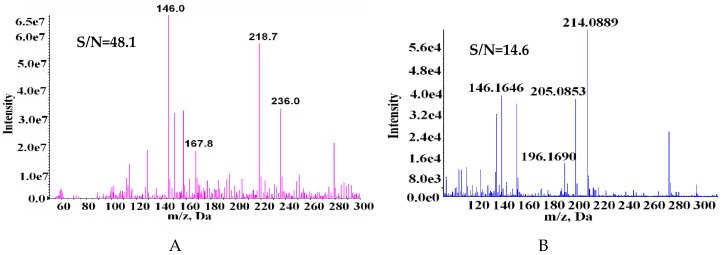
The full scan mass spectra of 10 mg/L SPD by (**A**) DART-MS; (**B**) UHPLC-ESI-QTOF method. S/N was given to ion at *m/z* 146.

An ultra-high performance liquid chromatography liquid chromatograph coupled with electrospray ionization quadrupole time-of-flight mass spectrometry (UHPLC-ESI-QTOF) has been demonstrated to provide high separation capacity and accurate mass, which facilitates the elucidation of the fragmentation schemes of target compounds [15]. To assess a practicable way of quantifying SPD in food by the DART-MS method, the results obtained by DART-MS were comprehensively compared with those obtained by the UHPLC-ESI-QTOF method. Since the positive ion mode of SPD can provide higher signal intensity than that of negative ions (data not shown), all the following experiments used positive detection mode. We first compared the full scan mass spectra of SPD and then compared its MS/MS spectra at the same concentration (10 µg/mL). In the full scan MS spectrum of DART-MS (Figure 1A), the observed MS pattern was the parent ion at *m/z* 146 along with several ion adducts in the background (Figure 1A). The presence of *m/z* 218.7 identified as [SPD + C_2_H_4_ + HCOOH]^+^, and *m/z* 236 may be derived from [SPD + H + 2 COOH]^+^. The ion at *m/z* 218.7 may be obtained by oxidation of the methanol added to the SPD with an ethylene molecule, and ion at *m/z* 236 may be the ion at *m/z* 146.0 adding two carboxylate ions. In the UPLC-QTOF-MS (Figure 1B), the precursor ion *m/z* 146 was also observed, along with a different background from DART-MS. The *m/z* 205 ion may be the product ion at *m/z* 146 plus a CH_3_COOH molecule; the ion at *m/z* 214 was tentatively identified as [SPD + HCHO + K^+^]^+^. The results show that the measured MS spectra are differ between the DART-MS and UHPLC-ESI-QTOF methods. Adduct ions with ammonia and formic acid derived from the atmosphere or solvent are usually found in the DART-MS method, while Na^+^ and K^+^ adduct ions usually occur with the UHPLC-ESI-QTOF method. Besides, the signal to noise (S/N) ratio of DART-MS is higher than that of UHPLC-ESI-QTOF.

**Figure 2:**
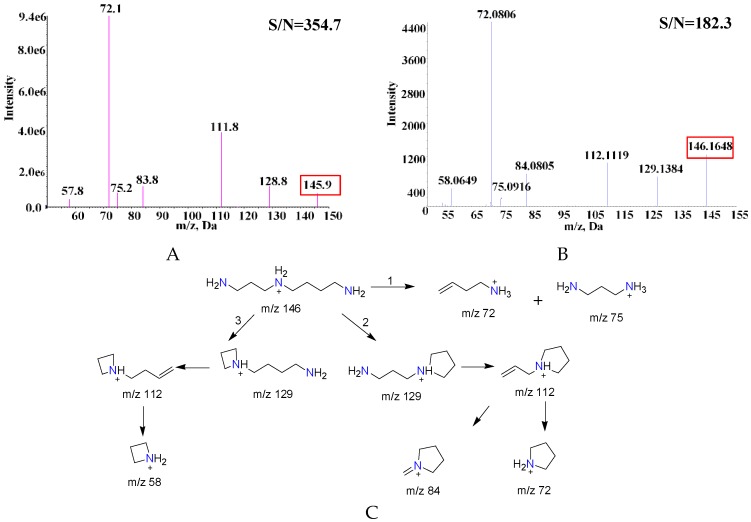
The MS/MS spectra of SPD in (**A**) DART-MS; (**B**) UHPLC-ESI-QTOF; and (**C**) proposed fragmentation scheme of SPD.

The MS/MS spectra obtained by both these two methods are shown in Figure 2. There is no obvious difference in the MS/MS spectra between the DART-MS (Figure 2A) and UHPLC-ESI-QTOF method (Figure 2B). According to the accurate mass by UHPLC-ESI-QTOF using a SPD solution, the possible fragmentation of SPD is proposed in Figure 2C. The results showed that there might be three different pathways in the SPD fragmentation. The first pathway is to obtain ions of *m/z* 75 and *m/z* 72 when the C–N bond of *m/z* 146 is broken; then, after the cleavage of the C–N bond from the *m/z* 146 species, the ions at *m/z* 75 and *m/z* 72 were obtained. The second cleavage pathway is *m/z* 146 undergoing an intramolecular deamination to form *m/z* 129 ion fragments, followed by loss of a neutral ammonia molecule to obtain *m/z* 112 fragment ions, and subsequent losses of C_2_H_2_ and CH_3_C≡CH to produce the fragment ions at *m/z* 84 and *m/z* 72, respectively. The third is the loss of a CH_2_=CH–CH=CH_2_ molecule from ion at *m/z* 112 to form an ion at *m/z* 58.

### 2.2. Optimization of the DART-MS Conditions

#### 2.2.1. Effect of Gas Heating Temperature of DART Ion Source

**Figure 3:**
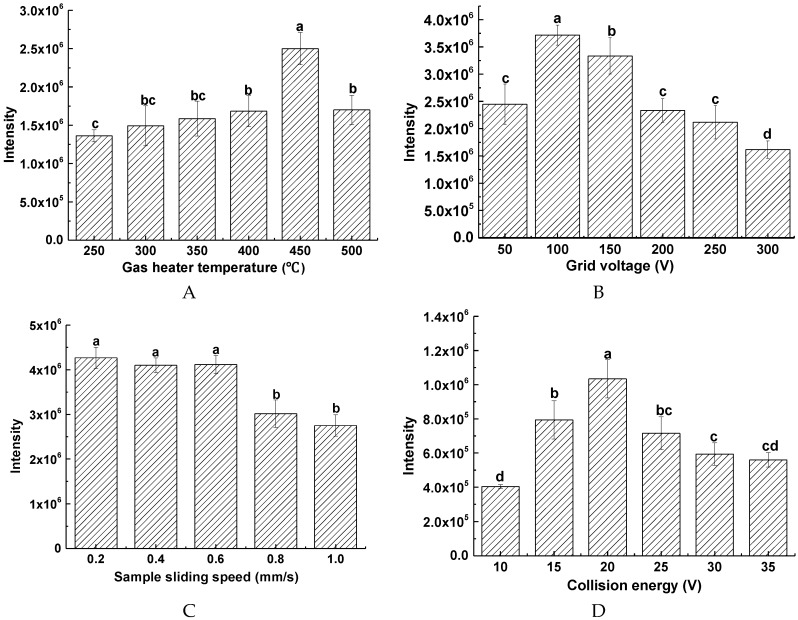
Optimization of determination conditions of SPD by DART-MS/MS: (**A**) gas temperature; (**B**) grid voltage; (**C**) sample sliding speed; (**D**) collision energy. Bars with different alphabets indicate significant differences between the mean values (*p* < 0.05), while the same letters indicate no significant differences between the means values (*p* > 0.05).

The gas temperature is an important ionization parameter in the analysis of SPD by DART-MS. The response intensity of SPD parent ion at *m/z* 146 was measured at different temperatures ranging from 250 °C to 500 °C. The relationship between the temperature and the response intensity of *m/z* 146 is shown in Figure 3, where the intensity of *m/z* 146 increased with increasing temperature near 450 °C, after which, the intensity started to decrease drastically beyond 450 °C. Therefore, 450 °C was selected as the gas heating temperature during the analysis.

#### 2.2.2. Effect of Grid Voltage

The grid voltage can perform a variety of functions, including acting as an ion repeller removing ions of opposite polarity, thus preventing the loss of signal caused by the recombination among of these ions [13]. Furthermore, it has a significant effect on eliminating reactive ions such as NO^+^ in the atmosphere and reducing the chemical background [16]. As seen in Figure 3B, a noticeable increase in the response intensity of *m/z* 146 was observed as the temperature was increased from 50 to 100 V, and the intensity was the highest at 100 V. The study by Harris also showed that the grid voltage may affect the sensitivity of the analysis [17], and the sensitivity of the protonated ions will be greatly improved at the lower grid voltage. Based on these results, grid voltage of 100 V was chosen for further analysis.

#### 2.2.3. Effect of Sample Presentation Speed

On the DART ion source, the sample was introduced into the mass spectrometer through a glass bar placed on a linear track. Therefore, the response intensity of *m/z* 146 can be affected by the sliding speed of the sample glass. The response intensity of *m/z* 146 was measured and the results are shown in Figure 3C. In the range of 0.2–0.6 mm/s, there is no significantly difference in the response intensity. However, when the speed exceeds 0.6 mm/s, the response intensity of *m/z* 146 gradually decreases with increasing speed. Finally, the sample sliding speed is maintained at 0.6 mm/s. 

#### 2.2.4. Effect of Collision Energy (CE)

The CE significantly affects the response intensity of the daughter ion at *m/z* 72. In this study, the CE was optimized from 10 to 35 V (Figure 3D). The results showed that with the increase of the CE, the response intensity of *m/z* 72 first increased and then decreased. At the CE of 20 V, the *m/z* 72 gave the highest response intensity. Accordingly, the CE was set to 20 V in the further studies.

#### 2.2.5. Method Validation

It is necessary to validate any newly developed analysis method. Both the DART-MS and UHPLC-ESI-QTOF methods exhibition good linearity, and the measured recoveries, LOD, LOQ, and precision are shown in Table 1. In the DART-MS method, the higher correlation coefficient (R^2^ = 0.990) showed that the linear relationship is excellent. Although there is no chromatographic separation, the LOD of SPD (0.025 mg/kg) measured by DART-MS method was significantly lower than that of UHPLC-ESI-QTOF method (0.036 mg/kg). The intra-day precision of SPD was calculated by repeated determination of standard solution six times in the same day. The intra-day and inter-day precisions were 3.60% and 7.50%, respectively. While in the UHPLC-ESI-QTOF method, the intra-day and inter-day precisions were 2.15% and 5.30%, respectively. Therefore, the reproducibility of the DART-MS method is superior to the literature, which in soybeans ranged from 5 to 38% by HPLC-FD method [18]. However, it is slight lower than that of UHPLC-ESI-QTOF method. The recoveries of SPD measured by DART-MS ranged from 102.79% to 148.44%, and the recoveries of SPD measured by UHPLC-ESI-QTOF ranged from 95.64% to 103.60% (Table 2). All these results demonstrated that DART-MS has a satisfactory reproducibility.

### 2.3. Matrix Effect

The matrix effect generally occurs during the ionization of analyte, which can adversely affect the quantitative analysis of the target. In this experiment (Table 3), the No. 12 variety with the highest SPD content was selected for investigation of matrix effect. A series of SPD solution was prepared with 80% methanol as a blank matrix sample (0.312–10 µg/mL). The matrix effect of the No. 12 variety measured by DART-MS and UHPLC-ESI-QTOF method were 13.20% and 20.16%, respectively. The positive value of matrix effect indicating that the sample matrix may cause a slight enhancement of SPD ionization. Depending on the matrix effect ≤20%, there may be a slight matrix effect in these methods [19]. Unexpectedly, the matrix effect of the DART-MS method is lower than that of the UHPLC-ESI-QTOF method, although there is no chromatographic separation process in the DART-MS method. A possible reason is the difference between the ionization mechanisms of DART-MS and UHPLC-ESI-QTOF.

### 2.4. Qualitative Analysis of SPD in Beans by DART-MS

The spermidine contents were quantified from the peak areas using the multiple reaction monitoring mode (ion pairs *m/z* 146 - 72) both in DART-MS/MS (+) versus the UHPLC-ESI-QTOF (+) method. As shown in Table 4, the SPD of the 12 cultivars ranged from 2.01 mg/kg (No.9) to 12.08 mg/kg (No. 12). The results obtained by DART-MS are in good agreement with those from UHPLC-ESI-QTOF, suggesting that the former could be used as a rapid alternative (~1.3 min per three samples) to classic UHPLC-ESI-MS method (~15 min per three samples) (see Supplementary Figure S1). 

The SPD concentration ranges between 88 and 389 mg/kg dry soybeans according to different investigations [18], which was significantly higher than our finding. The genotype, soil fertility and planting conditions may impact the SPD content of beans.

In order to evaluate the feasibility of rapid quantitative analysis of SPD in food samples by the DART-MS/MS method, the measured results were compared with those obtained by UHPLC-ESI-QTOF. As shown in Table 4, the data of barley and beans samples obtained by DART-MS are in good agreement with the results from UHPLC-ESI-QTOF. However, the validation parameters of the UHPLC-ESI-QTOF method (Table 1) indicates that precision of intraday and interday were slightly better than those found for the DART-MS method, although it is acceptable for high-throughput analysis. Moreover, the RSD value of the former is significantly higher than that of the latter, which may be because the former is determination in an open space, some oxygen anions and hydrated ions may compete the ionization process of the SPD. The LOD, LOQ, and recovery for the SPD measured by DART-MS were superior to those of UHPLC-ESI-QTOF. Although the intraday and interday precision of DART-MS is slightly lower than that of the UPLC-QTOF method, which is acceptable for high throughput analysis. The result showed that DART-MS technology provides a fast and reliable analytical method for determining the content of SPD in beans.

## 3. Materials and Methods

### 3.1. Sample and Reagents

**Figure 4:**
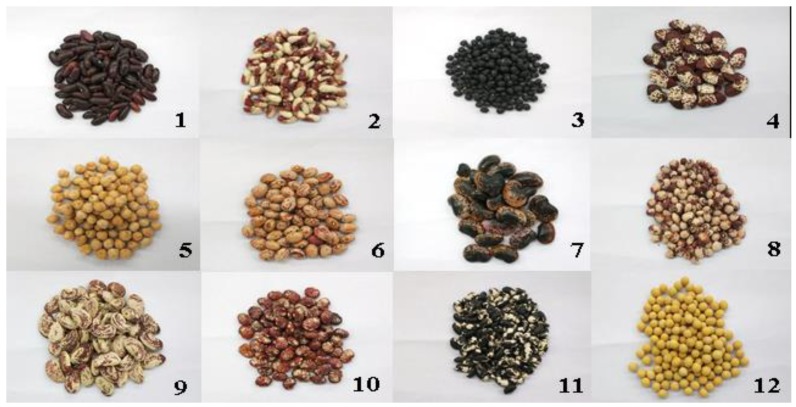
Representative photographs of twelve bean cultivars.

Twelve bean varieties of cultivated in China were purchased from a local supermarket in Chengdu, China in September 2017 (Figure 4). The whole beans were ground into powders and passed through a 60-mesh sieve. The flours was stored at −18 °C prior to analysis.

Acetonitrile and menthol (HPLC grade) was purchased from Sigma (St.Louis, MO, USA). Formic acid (HPLC grade) was purchased from Tianjin Kemiou Chemical Reagent Co., Ltd. (Tianjing, China) SPD standards (≥98%) purchased from Shanghai Yuanye Biotechnology Co., Ltd. (Shanghai, China).

### 3.2. Preparation of Standard and Sample Solution

Samples of beans (1.000 g) were weighed in a 50 mL centrifuge tube, 80% methanol (10 mL) was added and the mixture vortexed for 2 min. The mixtures were then sonicated in an ultrasonic bath for 20 min. After centrifugation of the extract, the supernatant was filtered and the extract was stored at −18 °C for further analysis. Working standard solutions of SPD ranging from 0.312 mg/L to 10 mg/L were prepared by diluting the SPD stock standard solution (20.0 mg/mL) with 80% menthol (*v/v*). All of standard solutions were stored at −18 °C before further analysis.

### 3.3. DART-MS Analysis 

The DART-MS analysis system comprised a DART ion source (DART-SVP, IonSense, Saugas, MA, USA) connected to a 3500 triple quad mass spectrometer (SCIEX, Concord, ON, Canada). SPD or sample solutions (2.0 µL) were deposited on the tips of glass sticks. These sticks were held on a linear rail, and then passed through the gap to introduce the sample ions into the mass spectrometer. The optimized DART settings were as follows: the distance between the DART ion source and the ion transfer tube was 1.0 cm; positive ion mode; gas temperature 450 °C; and grid electrode voltage 100 V. A constant speed of 0.6 mm/s was used for the Dip-It tip rail system. High-purity helium (99.999%) was used as the running gas.

### 3.4. UHPLC-QTOF-MS Analysis 

A Nexera X2 UHPLC system (Shimadzu, Kyoto, Japan) was employed for analyte separation. The system was equipped with a vacuum degasser, auto sampler, pump and PDA detector. The extracts were analyzed on a C18 column (2.1 mm i.d × 100 mm, 1.6 μm particle size, Phenomenex, Torrance, CA, USA). Mobile phase A was acetonitrile, and mobile phase B was ultrapure water with 0.2% formic acid solution. According to a previously described method [20] and slightly modified, the elution gradient is: initial concentration is 90% B; 0–2 min, 90–50% B; 2–3 min, 50–0% B; 3.1 min, 90% B, 3.1–5 min, keeping 90% B, total run time is 5 min, flow rate is 0.20 mL/min, injection volume is 2 µL, and oven temperature 40 °C. The high resolution mass spectral of the analytes was obtained using an X500R tandem mass spectrometer (SCIEX, Concord, ON, Canada). The quantitative detection of SPD in positive ionization mode and multiple reaction monitoring (MRM) acquisition. The mass scan range is *m/z* 50–400 Da and the scan time is 0.1 s The mass condition was: ion source gas 1 and gas 2, 45 psi and 50 psi; curtain gas 35 psi; declustering potential 60 V; collision energy 25 V.

### 3.5. Method Validation

In this study, the reliability of the method was verified by measuring spike recovery, mechanistic effects, limit of detection (LOD) [15], limit of quantitation (LOQ), intraday and interday precision. In this experiment, we selected the No. 12 variety with the highest SPD content for method validation. The LOD and the LOQ were determined by measuring the concentrations corresponding to SPD at signal-to-noise ratios (S/N) of 3 and 10, respectively. Then, the spiked recoveries were determined by analyzing three spiked concentrations of 0.625, 1.25, and 2.5 mg/mL. The volume ratio of the sample to the spiked amount is 1:1. The spike recovery was calculated using [21].

The standard curve of the matrix effect was made by serially diluting a series of standard solutions (10 mg/L) with the sample solution, and an equivalent concentration of the standard solution was diluted with 80% methanol as a blank. The calibration curve ranged from 0.312 mg/mL to 10 mg/mL. The matrix effect is calculated by the following formula [22,23]:

(1) Matrix effect (%) = (a−b)/a × 100% 

where a is slope of matrix calibration curve, where b is slope of solvent calibration curve.

### 3.6. Data Analysis

All experiments were repeated six times. The results were mean ± standard deviation, and the significant difference was determined at 95% confidence interval by analysis of variance followed by Duncan’s least significant test. 

## 4. Conclusions

In summary, DART-MS was demonstrated to be a powerful analytical technique for the rapid quantitative analysis of SPD in beans. Good agreement between DART-MS and UHPLC-ESI-QTOF results was obtained for 12 bean samples, ng that the former could be used as a rapid alternative (~1.3 min per three samples) to conventional UHPLC-ESI-QTOF analyses (~15 min per three samples). This study indicated that DART-MS will greatly simplify the SPD analysis period due to the elimination of tedious derivatization steps and lengthy separation processes. The method is environmentally friendly due to the fact it does not use a toxic mobile phase. Furthermore, the results of this study showed that the bean varieties provide a good source of SPD.

## Figures and Tables

**Table 1:** The validation parameters of DART-MS versus UHPLC-ESI-QTOF method.

Method	Linear	R^2^	LOD (mg/kg)	LOQ (mg/kg)	Precision	
					Intra-Day	Inter-Day
DART-MS	y = 292923x − 45007	0.990	0.025	0.078	3.6%	7.50%
UHPLC-ESI-QTOF	y = 89517x − 8931	0.999	0.036	0.131	2.15%	5.30%

**Table 2:** The spiked recovery of DART-MS versus UHPLC-ESI-QTOF method.

Variety	Sample Measurement (mg/L)	Spiked (mg/L)	DART-MS Recovery (%)	UHPLC-ESI-MS Recovery (%)
NO.12	1.20 ± 0.03	0.625	102.79 ± 3.13	95.64 ± 1.44
		1.25	115.70 ± 4.01	93.31 ± 1.91
		2.5	148.44 ± 3.83	103.60 ± 1.75

**Table 3:** Matrix effects of the No.12 variety by DART-MS versus UHPLC-ESI-QTOF method.

Matrix	Method	Equation of Calibration Curve	Matrix Effect (%)
Blank	DART-MS	y = 292923x − 45007	-
	UHPLC-ESI-QTOF	y = 89517x − 8931	-
NO. 12	DART-MS	y = 331591x + 26566	13.20%
	UHPLC-ESI-QTOF	y = 107561x + 528890	20.16%

**Table 4:** The SPD content of beans by DART-MS versus UHPLC-ESI-QTOF methods (average ± SD).

Number	DART-MS (mg/kg)	UHPLC-ESI-QTOF (mg/kg)
1	3.42 ± 0.05	3.38 ± 0.06
2	3.87 ± 0.17 *	3.19 ± 0.08
3	9.62 ± 0.15 *	9.16 ± 0.10
4	10.44 ± 0.26 *	9.55 ± 0.14
5	5.28 ± 0.28	4.90 ± 0.10
6	3.58 ± 0.23 *	5.22 ± 0.08
7	2.96 ± 0.10 *	2.15 ± 0.07
8	3.63 ± 0.14 *	1.95 ± 0.03
9	2.01 ± 0.06 *	1.84 ± 0.01
10	5.11 ± 0.31 *	3.34 ± 0.20
11	4.55 ± 0.22 *	3.64 ± 0.10
12	12.08 ± 0.35 *	13.45 ± 0.19

The number in the table corresponds to the bean sample number in Figure 4. * Means significant difference (*p* < 0.05).

## Supplementary Materials

Figure S1: MRM chromatograms of (A) 10 mg/L SPD standard and (B) NO.12 sample in DART-MS, n = 3; (C) 10 ppm SPD standard in and (D) NO.12 sample in UHPLC-ESI-QTOF, n = 1. Figure S2: The representative MS/MS spectra of NO.12 sample in (A) DART-MS, and in (B) UHPLC-ESI-QTOF.
