# Supplementary material for: Rapid Evaluation of Spermidine from 12 Bean Cultivars by Direct Real-Time Mass Spectrometry Analysis
Source: Molecules. 2018 Aug 25;23(9):2138. doi: 10.3390/molecules23092138 (PMC6225280; doi:10.3390/molecules23092138)
Supplement: Supplementary file 1 [file molecules-23-02138-s001.pdf]

Rapid Evaluation of Spermidine from 12 Bean Cultivars by Direct Analysis in Real-Time Mass Spectrometry

Tao Wu [1], Xiaoyu Wu [1], Xv Yuan [1], Yi Wang [2], Wenhua Zhou [3] and Weili Li [1,4,*]

[1] School of Food and Biotechnology, Xihua University, No. 9999 Hongguang Avenue, Chengdu 610039, China; wutao@mail.xhu.edu.cn (T.W.); wxy501267118@126.com (X.W.); suju980802948@126.com (X.Y.)
[2] Xi'an Manareco New Materials Co. Ltd., Xi'an 710077, China; wang.yi@xarlm.com
[3] Key Laboratory of Processed Food for Special Medical Purpose, Central South University of Forestry and Technology, No. 498 Shaoshan Road, Changsha 410004, China; zhowenhua@126.com
[4] College of Food Engineering and Biotechnology, Tianjin University of Science and Technology, Tianjin 300000, China
* Correspondence: liweli1207@126.com; Tel./Fax: +86-28-8772-0552

Received: 24 July 2018; Accepted: 20 August 2018; Published: date

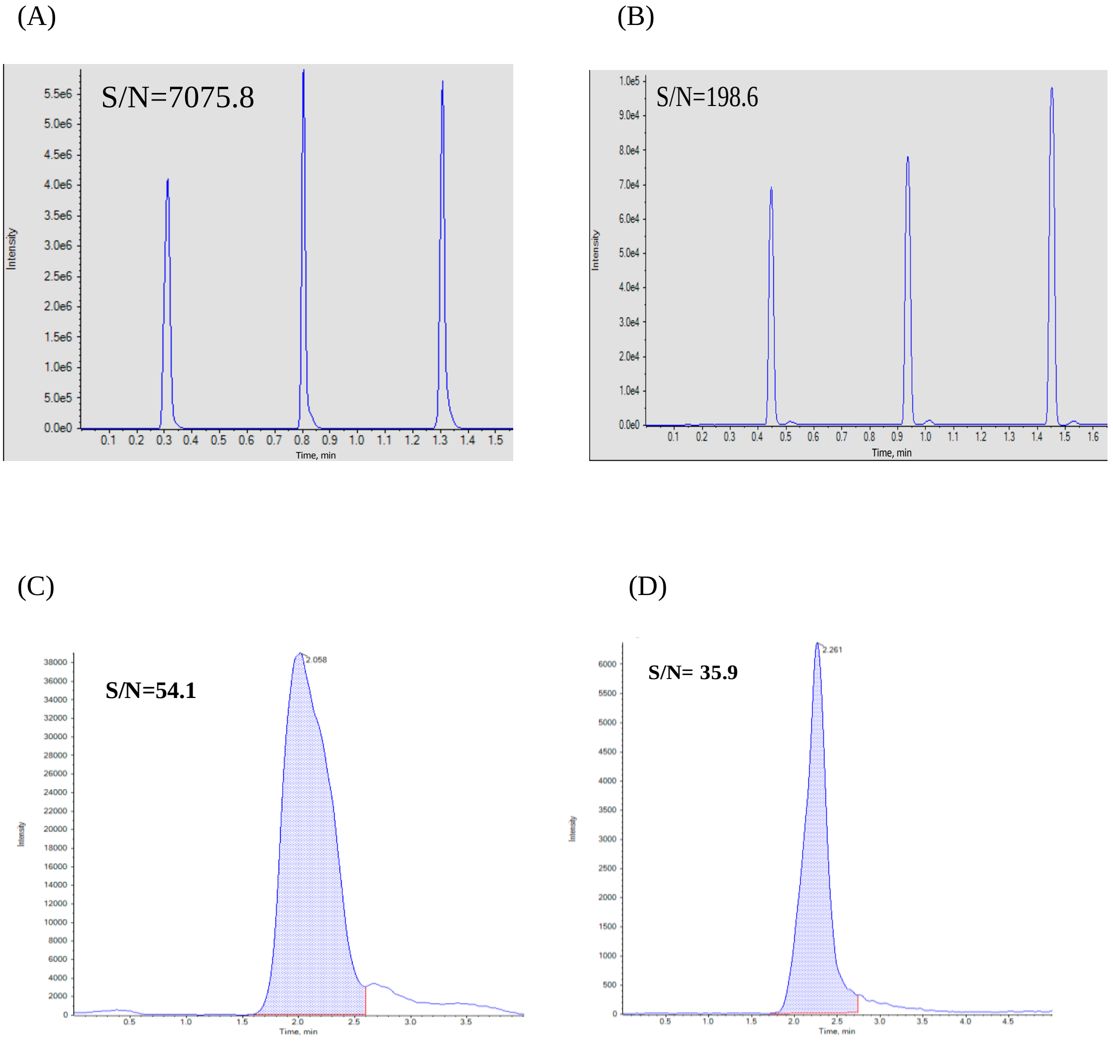

Figure S1: MRM chromatograms of (A) 10 mg/L SPD standard and (B) NO.12 sample in DART-MS, n=3; (C) 10 ppm SPD standard in and (D) NO.12 sample in UHPLC-ESI-QTOF, n=1.

(A) (B)

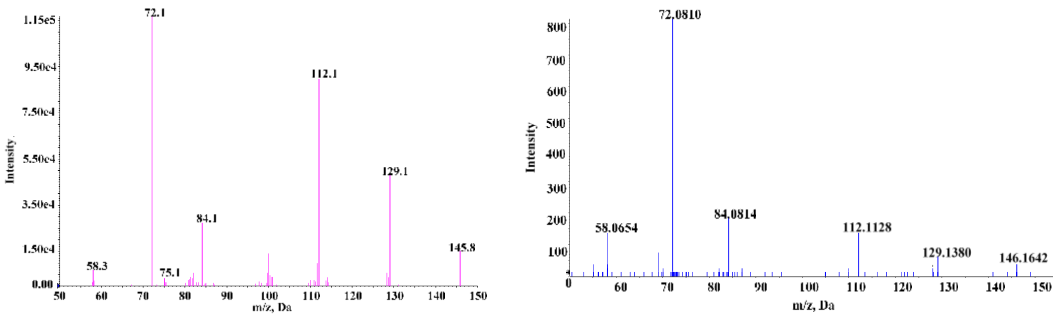

Figure S2: The representative MS/MS spectra of NO.12 sample in (A) DART-MS, and in (B) UHPLC-ESI-QTOF.
